# Neck Circumference is an Effective Supplement for Nonalcoholic Fatty Liver Disease Screening in a Community-Based Population

**DOI:** 10.1155/2020/7982107

**Published:** 2020-05-16

**Authors:** Chaohui Jian, Yiting Xu, Xiaojing Ma, Yun Shen, Yufei Wang, Yuqian Bao

**Affiliations:** Department of Endocrinology and Metabolism, Shanghai Jiao Tong University Affiliated Sixth People's Hospital, Shanghai Clinical Center for Diabetes, Shanghai Key Clinical Center for Metabolic Disease, Shanghai Diabetes Institute, Shanghai Key Laboratory of Diabetes Mellitus, Shanghai 200233, China

## Abstract

**Background:**

Accumulating evidence has shown that neck circumference (NC) is associated with obesity-related metabolic abnormalities. Nonalcoholic fatty liver disease (NAFLD) is regarded as a liver manifestation of metabolic syndrome. This study aimed to investigate the relationship between NC and liver fat content (LFC) and NAFLD.

**Methods:**

A total of 1698 subjects (577 men and 1121 women) from the Shanghai community were enrolled. All the subjects underwent NC measurement and biochemical measurements. LFC was calculated using the parameters from abdominal ultrasound images. Elevated NC was defined as NC ≥38.5 cm in men and NC ≥34.5 cm in women.

**Results:**

Subjects with NAFLD based on the LFC measurement had higher values of NC, liver enzyme profiles, homoeostasis model assessment-insulin resistance index, and LFC than those without NAFLD (all *P* < 0.05), irrespective of sex. NC showed an upward trend with the increase of LFC in both men and women (both *P* < 0.05). An elevated NC could identify 55.22% of men and 50.29% of women with NAFLD based on quantitative ultrasonography. The positive correlation between NC and LFC remained significant even after adjustment for central obesity (both *P* < 0.05). After adjusting for confounding factors, the risk of NAFLD in subjects with an elevated NC was 1.52-fold higher in men (*P*=0.036) and 2.31-fold higher in women (*P* < 0.001).

**Conclusions:**

There was a significant and positive correlation between NC and LFC. The risk of NAFLD increased significantly in subjects with an elevated NC.

## 1. Introduction

Nonalcoholic fatty liver disease (NAFLD) is an obesity-related metabolic disease, which has now become the leading cause of chronic liver diseases worldwide [1]. In recent years, the prevalence of NAFLD in China has been increasing. According to the latest report in 2019, the prevalence of NAFLD in China has reached 29.81% and exceeds 50% in overweight/obese subjects, even reaching up to nearly 80% [2, 3]. Due to its tight connection with insulin resistance (IR), NAFLD is considered to be a risk factor for future development of metabolic syndrome and its complications (such as type 2 diabetes mellitus and cardiovascular diseases) [4]. Furthermore, cardiovascular diseases are the leading cause of death in NAFLD patients. Early detection and intervention can reduce adverse outcome events. At present, the most commonly used method for NAFLD diagnosis is ultrasonography. Finding simpler and more effective indicators is of great significance for the prevention and control of cardiovascular diseases and adverse hepatic events in community-based populations.

Neck circumference (NC) is the girth below the thyroid cartilage protrusion. The NC measurement is easy and highly reproducible and has little variation. NC reflects ectopic fat deposition in the neck and is an important anthropological index for determining the degree of obesity, especially upper body obesity. Increasing evidence has shown that NC is associated with obesity-related metabolic abnormalities, such as metabolic syndrome, IR, and cardiovascular atherosclerosis [5–8]. Given that NAFLD is a risk factor for metabolic syndrome and its complications, several studies assessed the association between NC and NAFLD and proposed that NC was a simple predictor of NAFLD [9–11]. However, the diagnosis of NAFLD in previous studies was based on qualitative ultrasonography or the calculation of laboratory indicators (such as the fatty liver index, known as FLI) rather than on quantitatively assessing liver fat content (LFC).

Our previous study obtained NC cutoff points for the identification of metabolic syndrome by using magnetic resonance imaging to assess central obesity accurately in a community-based population [12]. Therefore, this study aimed to assess LFC through quantitative ultrasonography and determine the relationship between NC and LFC and the utility of NC cutoff points for the identification of NAFLD.

## 2. Materials and Methods

### 2.1. Subjects

A total of 1698 subjects (577 men and 1121 women) from the Zhabei community of Shanghai were enrolled from 2015 to 2016. A questionnaire survey, including a history of current and past diseases, medication, smoking, menopausal status, family diseases, and personal habits, was performed by well-trained investigators. The study was approved by the Ethics Committee of Shanghai Jiao Tong University Affiliated Sixth People's Hospital, and all subjects provided informed consent. Subjects with positive hepatitis B surface antigen or anti-hepatitis C virus antibody, excessive alcohol consumption in the past 12 months (≥210 g per week for men and ≥140 g per week for women), autoimmune liver disease, neck malformation or surgery history, thyromegaly, thyroid dysfunction, a valid history of cardiovascular diseases, tumors, severe liver and kidney dysfunction, acute infection, or current use of glucocorticoids or thyroid hormones, and those who were pregnant were excluded.

### 2.2. Anthropometric and Biochemical Measurements

All participants underwent physical examinations, and height, weight, NC, waist circumference (WC), and blood pressure were measured. The standard methods for all the anthropometric measurements were described in a previous study [12]. Body mass index (BMI) = weight (kg)/height^2^ (m^2^).

All subjects underwent examinations in the morning after a 10 h overnight fast. Individuals without a valid diabetic history took a 75 g oral glucose tolerance test, while anyone diagnosed as diabetes took the 100 g steamed bread meal test instead. Fasting and 2 h blood samples were collected. Measurements of fasting plasma glucose (FPG), 2-h plasma glucose (2hPG), glycated hemoglobin A1c (HbA1c), triglyceride (TG), total cholesterol (TC), low-density lipoprotein cholesterol (LDL-c), high-density lipoprotein cholesterol (HDL-c), C-reactive protein (CRP), fasting insulin, alanine aminotransferase (ALT), aspartate aminotransferase (AST), and gamma-glutamyl transpeptidase (GGT) were carried out based on standard methods as previously described [13]. The homoeostasis model assessment-insulin resistance index (HOMA-IR) was calculated as FPG (mmol/L) × fasting insulin (mU/L)/22.5.

### 2.3. Qualitative and Quantitative Diagnosis of NAFLD Based on Ultrasonography

All subjects received an abdominal ultrasonographic examination by a trained sonographer who was blind to the study design and clinical details of the participants using a Voluson 730 Expert B-mode ultrasonogram device (5.0-MHz transducer, GE Healthcare, Waukesha, WI, USA). A qualitative diagnosis of NAFLD was made.

The regions of interest in the images captured by the ultrasound device were analyzed using image software certified by the National Institutes of Health (ImageJ 1.41o, National Institutes of Health, Bethesda, MD, USA) [14]. All the instrument settings, including “gain,” “depth,” and “time-gain compensation,” were fixed for each measurement. A 3D abdominal organ-mimicking phantom (Model 057; Computerized Imaging Reference Systems, Norfolk, VA) was used for standardization of the ultrasound hepatic/renal echo-intensity ratio and hepatic echo-intensity attenuation rate. The LFC was then calculated based on the following equation: LFC (%) = 62.592 × hepatic/renal echo-intensity ratio + 168.076 × hepatic echo-intensity attenuation rate − 27.863 [14]. The quantitative diagnosis of NAFLD was defined as LFC ≥9.15% (expressed as LFC-NAFLD) [14].

### 2.4. Diagnosis Criteria

(1) As previously reported in our study, elevated NC was defined as an NC ≥38.5 cm in men and an NC ≥34.5 cm in women [12]. (2) According to the Chinese guidelines for the prevention and treatment of type 2 diabetes mellitus (2017 edition) [15], a WC ≥90 cm in men and a WC ≥85 cm in women were considered as central obesity. (3) When enrolled in this study, individuals who smoked at least one cigarette per day for more than six months were regarded as smokers [16].

### 2.5. Statistical Analysis

The statistical analysis was performed using SPSS 20.0 (IBM SPSS Inc., Chicago, IL, USA), and a two-tailed *P* < 0.05 was considered statistically significant. Normally distributed data, skewed data, and categorical variables are shown as the mean ± standard deviation, median (interquartile range), and number (percentages), respectively. The intergroup comparisons of the aforementioned variables were performed with student's *t* test, Wilcoxon rank sum test, and chi-squared test, respectively. A Spearman analysis was used to show the correlations between LFC and other variables. The associations between NAFLD and elevated NC were analyzed by logistic regression, and the independent correlations between NC and LFC were analyzed by multiple linear regression analysis in both men and women.

## 3. Results

### 3.1. Clinical Characteristics of the Study Participants

In the total population, the subjects, with an age range from 27 to 82, had a median age of 60 (55–65) years, a median NC of 34.5 (32.6–37.1) cm and a median LFC of 8.8 (6.8–24.3) %. The prevalence of NAFLD was 45.88% (779/1698) for the quantitative diagnosis based on the LFC measurement and 38.52% (654/1698) for the qualitative diagnosis. The levels of NC and LFC in the LFC-NAFLD group were higher than those in the non-LFC-NALFD group in both men and women (all *P* < 0.05). Subjects with LFC-NAFLD had higher values of BMI, WC, blood pressure, liver enzyme profiles, blood glucose, HbA1c, fasting insulin, TG, LDL-c, CRP, and HOMA-IR and lower levels of HDL-c (all *P* < 0.05; Table 1), irrespective of sex. In addition, there were higher values of WC, NC, blood pressure, ALT, and GGT and lower levels of TC, HDL-c, LDL-c, and LFC in men than in women (all *P* < 0.05; Table 1).

### 3.2. The Association of NC and LFC Levels

**Figure 1:**
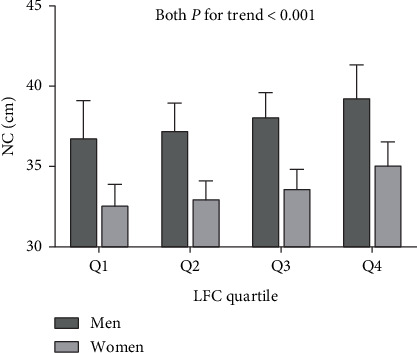
Neck circumference (NC) levels according to liver fat content (LFC) quartile.

Subjects were divided into four groups according to quartiles of LFC levels: Q1 (LFC <6.8%), Q2 (6.8% ≤ LFC <8.8%), Q3 (8.8% ≤ LFC <24.3%), and Q4 (LFC ≥24.3%). From Q1 to Q4, the levels of NC were 36.8 cm, 37.3 cm, 38.1 cm, and 39.3 cm in men and 32.6 cm, 33.0 cm, 33.7 cm, and 35.1 cm in women, respectively, which demonstrated that NC presented a significant upward trend with the increase of LFC in both men and women (both *P* for trend <0.001; Figure 1).

### 3.3. Identification of NAFLD by NC Cutoff Points

**Figure 2:**
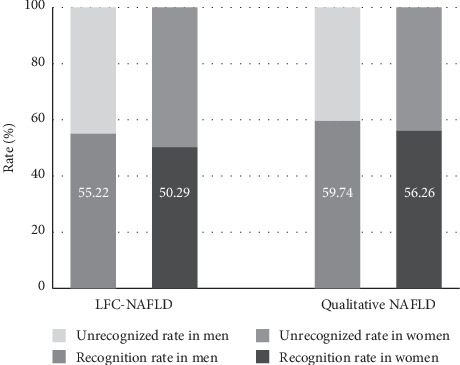
The identification of neck circumference (NC) cutoff points for nonalcoholic fatty liver disease (NAFLD) based on quantitative and qualitative ultrasonography.

The prevalence of LFC-NAFLD in men was 46.45% (268/577), and 148 cases could be identified by the NC cutoff point, with a recognition rate of 55.22%. The prevalence of LFC-NAFLD in women was 45.58% (511/1121), and 257 cases could be identified by the NC cutoff point, with a recognition rate of 50.29%. The prevalence of NAFLD based on the qualitative measurement in men was 40.03% (231/577), and the recognition rate with the NC cutoff point reached 59.74% (138/231). The prevalence of NAFLD based on the qualitative measurement in women was 37.73% (423/1121), and the recognition rate with the NC cutoff point was 56.26% (238/423). There was no sex difference in the recognition rates with NC cutoff points for NAFLD based on either the quantitative or qualitative measurement (*P*=0.200, 0.409; Figure 2).

### 3.4. The Association of NC and NAFLD

There were significant and positive correlations between NC and LFC in both men and women (standardized *β* = 0.354 and 0.435, respectively, both *P* < 0.001). The multiple linear regression analysis in Table 2 showed that NC was an independent determinant factor of LFC after adjustment for age, blood pressure, HbA1c, HOMA-IR, lipid profiles, and CRP (standardized *β* = 0.195 and 0.228 for men and women, respectively, both *P* < 0.001). The positive correlation remained significant even after further adjustment for central obesity (standardized *β* = 0.111 and 0.126 for men and women, respectively, both *P* < 0.05).

**Figure 3:**
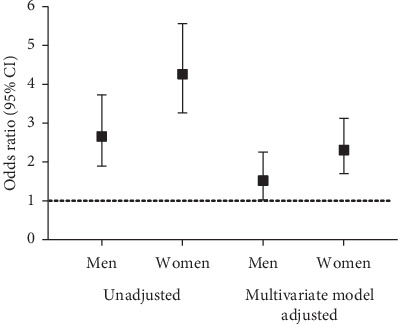
The association of neck circumference (NC) with risks of nonalcoholic fatty liver disease (NAFLD) in different models.

As shown in Figure 3, using LFC-NAFLD as the dependent variable, the logistic regression analysis showed that NC was significantly and positively correlated with NAFLD after adjustment for age, blood pressure, HbA1c, HOMA-IR, lipid profiles, and CRP. The risk of NAFLD in subjects with an elevated NC was 1.52-fold higher in men (OR = 1.520 (1.028–2.250), *P*=0.036) and 2.31-fold higher in women (OR = 2.307 (1.702–3.127), *P* < 0.001) than in those with a normal NC.

## 4. Discussion

Our study found that NC showed an increasing trend with the increase of LFC in both men and women. There was still a significant and positive association between NC and LFC even after adjustment for confounding factors. NC cutoff points could effectively identify NAFLD whether on the basis of qualitative or quantitative measurement.

NAFLD, as a type of fatty liver, is regarded as the liver manifestation of metabolic syndrome [17]. It has been estimated that NAFLD will be the most frequent indicator for liver transplantation and regeneration in the coming decades [18]. NAFLD is followed by an increase in overall cardiovascular morbidity and mortality, which is significantly higher than that in those with extrahepatic malignant tumors and liver diseases [19]. Therefore, early diagnosis and effective management will improve prognoses and prevent secondary complications. NAFLD encompasses a broad clinical spectrum ranging from nonalcoholic fatty liver to nonalcoholic steatohepatitis, advanced fibrosis, cirrhosis, and finally hepatocellular carcinoma. Liver biopsy is the “gold standard” method for NAFLD diagnosis. However, it is not suitable for large-scale clinical applications due to its invasiveness.

As a simple anthropometric index for assessing upper body fat accumulation, the measurement of NC is simple and minimally affected by breathing and diet, with an explicit anatomic landmark, high repeatability, and low variability. Several studies have shown the association between NC and NAFLD. A cross-sectional study in China, with 2761 subjects, showed that NC was significantly and positively correlated with fasting insulin levels and HOMA-IR in both men and women. The prevalence of NAFLD presented an upward trend with the increase in the NC quartile, irrespective of central obesity. The optimal cutoff points of NC for NAFLD diagnosis were 37.25 cm for men and 32.90 cm for women [9]. Another multicenter cross-sectional study, which enrolled 2668 normal-weight subjects, showed that NC was elevated with increasing severity of NAFLD. NC was an independent risk factor for NAFLD in men. Taking the lowest quartile as a reference, after adjustment for confounding factors, the risks of NAFLD were 1.47- to 2.18-fold higher with the increase in the NC quartile [10]. In addition, Salmanroghani et al. assessed NAFLD in 590 inpatients using a semiquantitative method. The results showed that NC was significantly and positively correlated with NAFLD, NAFLD severity, and metabolic syndrome status. Multiple logistic regression analysis showed that after adjustment for age, BMI, WC, and waist-to-hip ratio, the higher NC quartiles resulted in a 1.24- to 3.23-fold increase in the risk of NAFLD. The optimal cutoff points of NC for NAFLD diagnosis were 39.25 cm for men and 34.85 cm for women [20]. The abovementioned studies arrived at different NC cutoff points due to ethnic differences in their study populations and different measurement methods and diagnostic standards for NAFLD.

The NC cutoff points we used in the current study were obtained from a previous study. We assessed central obesity using the precise standard-visceral fat area, which was measured with magnetic resonance imaging, and proposed that the optimal NC cutoff points for metabolic abnormalities were 38.5 cm for men and 34.5 cm for women [12]. In addition, our study assessed NAFLD using quantitative ultrasonography, which is a phantom-calibrated, computer-analyzed measurement, with 13% higher sensitivity, even up to 35% higher sensitivity in mild hepatic steatosis, and lower inter- and intraobserver variability than traditional qualitative measurements [21]. In our study, the prevalence of NAFLD based on the LFC measurement was 7.36% higher than that of the qualitative measurement. The current study proved that NC could identify NAFLD based on quantitative measurement. Elevated NC resulted in an additional 52% and 131% risk of NAFLD in men and women, respectively. Therefore, the measurement of NC is a simple but important supplement for the early screening of NAFLD in large-scale population studies.

The mechanism behind the association between NC and NAFLD remains unclear. On the one hand, upper body fat accumulation resulted in increased free fatty acid release [22, 23] and increased cytokines and adipocytokines production [24], which played an important role in the pathogenesis of NAFLD, namely, the “two hits” theory. Furthermore, the activation of carbohydrate response element binding protein and sterol regulatory element binding protein-1c upregulated glycolytic enzymes and fatty acid synthase to promote hepatic lipogenesis in obesity [25]. In addition, there was complex crosstalk between fat homeostasis and the liver regional immune system. Proinflammatory macrophages in neck adipose tissue decreased hepatocyte responsiveness to insulin by impairing insulin-mediated phosphorylation of insulin receptor substrate 1 (IRS1) and IRS1-associated PI3K in hepatocytes and produced high levels of neutrophil chemotactic proteins, contributing to increased hepatic neutrophil and macrophage infiltration and worsening liver damage [26].

There are several limitations in our study. First, this study was a single-center, community-based study, and the results should be further verified in multiple centers with a large-sample population. Second, in consideration of the cross-sectional study design, the utility of NC in predicting the development of NAFLD needs more prospective studies to be confirmed.

## 5. Conclusion

NC was significantly and positively associated with LFC in the Shanghai community of China. The risk of NAFLD was significantly higher in subjects with an elevated NC than in those with a normal NC. Therefore, the measurement of NC may be a simple but important supplement for NAFLD screening in large-scale population studies.

## Figures and Tables

**Table 1:** Characteristics of the study participants.

Variables	Non-LFC-NAFLD (*n* = 919)		LFC-NAFLD (*n* = 779)	
	Men (*n* = 309)	Women (*n* = 610)	Men (*n* = 268)	Women (*n* = 511)
Age (years)	63 (57–67)	59 (55–64)^*∗∗*^	61 (56–66)^#^	60 (56–64)^#^
BMI (kg/m^2^)	23.54 (21.81–25.25)	22.60 (20.82–24.50)^*∗∗*^	25.62 (24.03–27.59)^##^	25.36 (23.32–27.70)^##^
WC (cm)	84.0 (79.0–89.0)	78.0 (73.0–83.0)^*∗∗*^	91.0 (86.0–96.0)^##^	86.0 (81.0–92.0)^*∗∗*^^##^
NC (cm)	37.0 (35.2–39.0)	32.8 (31.3–34.1)^*∗∗*^	38.8 (37.1–40.4)^##^	34.5 (33.0–36.0)^*∗∗*^^##^
SBP (mmHg)	131 (120–142)	126 (114–138)^*∗∗*^	137 (127–149)^##^	132 (122–145)^*∗∗*^^##^
DBP (mmHg)	78 (72–84)	74 (68–81)^*∗∗*^	84 (77–90)^##^	78 (71–84)^*∗∗*^^##^
ALT (U/L)	18.0 (15.0–24.0)	16.0 (13.0–21.0)^*∗∗*^	23.5 (18.0–33.0)^##^	21.0 (16.0–28.0)^*∗∗*^^##^
AST (U/L)	20.0 (17.0–24.0)	20.0 (17.0–23.0)	21.0 (18.0–25.0)^##^	21.0 (18.0–25.0)^##^
GGT (U/L)	24.0 (19.0–33.0)	18.0 (15.0–26.0)^*∗∗*^	32.0 (24.3–43.8)^##^	25.0 (19.0–35.0)^*∗∗*^^##^
FPG (mmol/L)	5.77 (5.38–6.30)	5.65 (5.29–6.07)^*∗∗*^	6.00 (5.50–6.94)^##^	6.02 (5.57–6.72)^##^
2hPG (mmol/L)	7.35 (5.85–8.91)	7.04 (5.75–8.83)	8.33 (6.40–10.92)^##^	8.47 (6.78–11.28)^##^
HbA1c (%)	5.60 (5.40–5.90)	5.70 (5.40–6.00)^*∗*^	5.80 (5.50–6.30)^##^	5.80 (5.50–6.20)^##^
Fasting insulin (mU/L)	7.28 (4.97–9.96)	7.89 (6.02–10.46)^*∗∗*^	11.40 (8.09–16.18)^##^	12.15 (8.78–16.33)^##^
TC (mmol/L)	5.07 (4.53–5.60)	5.48 (4.88–6.27)^*∗∗*^	5.20 (4.55–5.86)	5.57 (5.01–6.26)^*∗∗*^
TG (mmol/L)	1.25 (0.88–1.78)	1.17 (0.89–1.67)	1.93 (1.36–2.78)^##^	1.66 (1.30–2.46)^*∗∗*^^##^
HDL-c (mmol/L)	1.32 (1.12–1.53)	1.58 (1.37–1.82)^*∗∗*^	1.15 (0.98–1.32)^##^	1.36 (1.18–1.57)^*∗∗*^^##^
LDL-c (mmol/L)	3.11 ± 0.75	3.36 ± 0.89^*∗∗*^	3.26 ± 0.80^#^	3.47 ± 0.83^*∗∗*^^#^
CRP (mg/L)	0.67 (0.32–1.46)	0.75 (0.36–1.34)	0.97 (0.58–1.71)^##^	1.30 (0.73–2.55)^*∗∗*^^##^
HOMA-IR	1.89 (1.30–2.63)	2.03 (1.47–2.78)^*∗*^	3.18 (2.09–4.59)^##^	3.35 (2.41–4.65)^##^
LFC (%)	6.5 (5.3–7.8)	7.1 (6.0–8.0)^*∗∗*^	24.2 (16.4–27.3)^##^	25.2 (17.2–33.6)^*∗*^^##^
Qualitative NAFLD, *n* (%)	0 (0)	0 (0)	231 (86.19)^##^	423 (82.78)^##^
Smoking, *n* (%)	130 (42.07)	6 (0.98)^*∗∗*^	102 (38.06)	3 (0.59)^*∗∗*^

Data are expressed as mean ± standard deviation for normally distributed variables or the median (interquartile range) for skewed distribution variables. ^*∗*^*P* < 0.05, men versus women in Non-LFC-NAFLD and LFC-NAFLD; ^*∗∗*^*P* < 0.01, men versus women in Non-LFC-NAFLD and LFC-NAFLD. ^#^*P* < 0.05, Non-LFC-NAFLD versus LFC-NAFLD in men and women; ^##^*P* < 0.01, Non-LFC-NAFLD versus LFC-NAFLD in men and women. LFC-NAFLD, nonalcoholic fatty liver disease based on liver fat content; BMI, body mass index; WC, waist circumference; NC, neck circumference; SBP, systolic blood pressure; DBP, diastolic blood pressure; ALT, alanine aminotransferase; AST, aspartate aminotransferase; GGT, gamma-glutamyl transpeptidase; FPG, fasting plasma glucose; 2hPG, 2-h plasma glucose; HbA1c, glycated hemoglobin A1c; TC, total cholesterol; TG, triglyceride; HDL-c, high-density lipoprotein cholesterol; LDL-c, low-density lipoprotein cholesterol; CRP, C-reactive protein; HOMA-IR, homoeostasis model assessment-insulin resistance index; LFC, liver fat content; NAFLD, nonalcoholic fatty liver disease.

**Table 2:** Linear regression analysis showing association of NC with LFC.

Variables	Men			Women		
	Standardized *β*	*t*	*P*	Standardized *β*	*t*	*P*
Unadjusted Model	0.354	9.090	<0.001	0.435	16.144	<0.001
Multivariate Model 1	0.195	4.929	<0.001	0.228	8.272	<0.001
Multivariate Model 2	0.111	2.593	0.010	0.126	4.220	<0.001

Multivariate Model 1 included age, NC, SBP, DBP, HbA1c, HOMA-IR, TG, HDL-c, LDL-c, and CRP. Multivariate Model 2 included age, NC, SBP, DBP, HbA1c, HOMA-IR, TG, HDL-c, LDL-c, CRP, and central obesity. NC, neck circumference; LFC, liver fat content; SBP, systolic blood pressure; DBP, diastolic blood pressure; HbA1c, glycated hemoglobin A1c; HOMA-IR, homoeostasis model assessment-insulin resistance index; TG, triglyceride; HDL-c, high-density lipoprotein cholesterol; LDL-c, low-density lipoprotein cholesterol; CRP, C-reactive protein.

## Data Availability

The datasets generated for this study will not be made publicly available because the ethical approval obtained for this study prevents the human data being shared publicly to protect patients' privacy. Requests to access the datasets should be directed to Yuqian Bao (yqbao@sjtu.edu.cn). This would be passed to the ethics committee who will decide whether they can access the data directly.
